# Circulating tumor DNA validity and potential uses in metastatic breast cancer

**DOI:** 10.1038/s41523-024-00626-6

**Published:** 2024-03-12

**Authors:** Ottavia Amato, Nefeli Giannopoulou, Michail Ignatiadis

**Affiliations:** 1 Department of Surgery, Oncology and Gastroenterology (DISCOG), University of Padova, Padova, Italy; 2 Medical Oncology 2, Istituto Oncologico Veneto IOV-IRCCS, Padova, Italy; 3 Department of Basic and Clinical Sciences, University of Nicosia Medical School, Nicosia, Cyprus; 4 Breast Medical Oncology Clinic, Institut Jules Bordet and Université Libre de Bruxelles, Brussels, Belgium

**Keywords:** Tumour biomarkers, Breast cancer

## Abstract

Following the first characterization of circulating tumor DNA (ctDNA) in the 1990s, recent advances led to its introduction in the clinics. At present, the European Society Of Medical Oncology (ESMO) recommendations endorse ctDNA testing in routine clinical practice for tumor genotyping to direct molecularly targeted therapies in patients with metastatic cancer. In studies on metastatic breast cancer, ctDNA has been utilized for treatment tailoring, tracking mechanisms of drug resistance, and for predicting disease response before imaging. We review the available evidence regarding ctDNA applications in metastatic breast cancer.

## Introduction

Tumor characterization has historically been based on tissue analysis, most often limited to a single biopsy of either the primary tumor or a metastasis. Recently, the discovery of nucleic acids of tumor origin in the bloodstream provided a different means of describing tumors’ molecular landscape. The term “liquid biopsy” refers to the analysis of neoplastic material isolated in blood or other fluids, shed by tumor cells residing in different body niches: the possible analytes include nucleic acids, circulating tumor cells, extracellular vesicles, tumor-educated platelets and proteins and metabolites^1,2^.

The presence of cell-free DNA (cfDNA) in plasma from healthy individuals, mainly shed from senescent haematopoietic cells, was first reported in 1948^3^. Only in the 1990s circulating tumor DNA (ctDNA) was characterized as an admixture of DNA released from cancer cells, representing the cfDNA fraction of tumor origin. At present, ctDNA testing is exploited in multiple research and clinical settings in oncology: as a tool for treatment tailoring and prognostication in the metastatic setting, detection of minimal residual disease after curative-intent therapy, and for cancer screening and diagnosis^1^. However, no single ctDNA assay appears suitable for all these purposes.

According to recent recommendations by the European Society of Medical Oncology (ESMO), there is sufficient evidence to support the routine use of ctDNA assays in clinical practice for tumor genotyping to direct molecularly targeted therapies in patients with metastatic cancer^4^. In fact, blood-based tumor genotyping may be more informative than tissue-based approaches in identifying molecular targets for precision medicine strategies, by reflecting variants from different disease sites, and might turn especially useful when rapid results are needed, by circumventing the delays and risks of realizing a tissue biopsy. Also, liquid biopsy can be easily performed serially, allowing to appraise temporal tumor heterogeneity and the emergence of resistance mechanisms. Nevertheless, tissue-based testing remains the gold-standard, also considering the limits of ctDNA assays in detecting genomic abnormalities other than single nucleotide variations (SNVs), such as gene fusions and copy number alterations. Moreover, a low fraction of cfDNA of tumor origin may lead to false-negative results, i.e. a variant present in the tumor cannot be detected in peripheral blood, while false-positive results may arise due to plasma DNA from apoptotic haematopoietic cells bearing stochastic somatic alterations, a phenomenon known as clonal haematopoiesis of indeterminate potential (CHIP)^4,5^. Thus, while the evidence supporting ctDNA assays to direct targeted treatments is now strong, their limitations should be taken into account, and a non-informative ctDNA test result should prompt reflex tissue-based testing^4^.

**Fig. 1:**
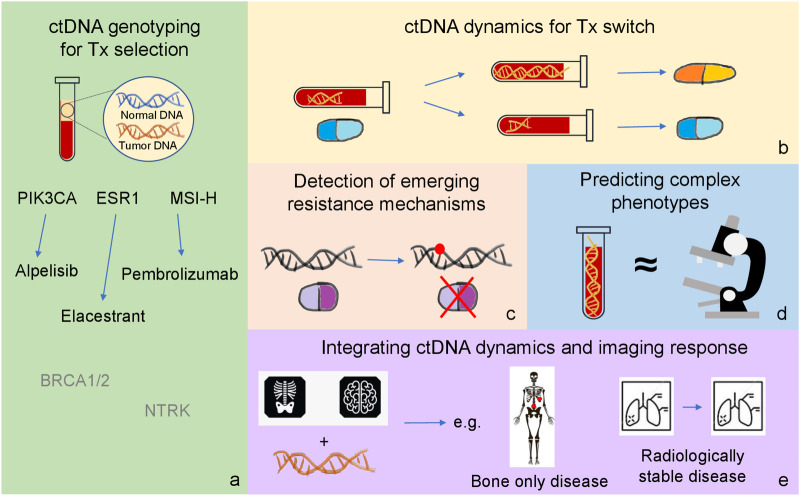
Current and future applications of ctDNA testing in metastatic breast cancer. The figure summarizes different clinical situations in which ctDNA is currently being exploited or might be exploited in the near future for the treatment of metastatic breast cancer: **a** treatment selection; **b** treatment switch; **c** detection of emerging resistance mechanisms; **d** prediction of complex phenotypes; **e** integration of ctDNA dynamics with imaging response criteria.

In this article, we will review available evidence supporting the use of ctDNA assays to direct clinical decisions in metastatic breast cancer (mBC) (Fig. 1).

## Tumor genotyping for treatment selection

### Molecular screening programs in mBC

Diverse studies across cancers explored the concept of sequencing tumor genomes to identify potential therapeutic targets^6^. The plasmaMATCH trial was a large prospective study evaluating the clinical validity and utility of ctDNA assessment in directing molecularly targeted treatments for mBC patients^7^. In this phase 2 trial, patients with mutations of *ESR1*, *ERBB2*, *AKT1* or *PTEN* identified on ctDNA were offered treatment with increased-dose fulvestrant, neratinib +/− fulvestrant, capivasertib + fulvestrant or capivasertib monotherapy, respectively, plus a fifth cohort enrolling patients with triple-negative mBC treated with olaparib + the ATR inhibitor AZD6738^7^. The primary endpoint was the overall response rate (ORR) in each cohort. The study not only showed that ctDNA testing is highly accurate and sensitive compared to tissue-based mutation testing, but also confirmed a relevant clinical activity of treatment strategies targeting rare driver mutations in mBC^7^.

Consistent results came from the SAFIR02-BREAST trial, suggesting that a precision medicine approach matching drugs to genomic alterations classified as level I or II according to the ESMO Scale of Actionability of Molecular Targets (ESCAT) improves outcomes for mBC patients. However, most of the enrolled patients were treated based on tissue genotyping, and ctDNA was an option in case of not feasible tissue biopsy^8^.

Recently, in a large trial investigating the clinical utility of ctDNA profiling for directing targeted treatments in advanced cancers, mBC was one of the five most represented neoplasms, with 171 mBC patients out of 1772 enrolled overall^9^. The authors demonstrated the feasibility of treatment matching based on ctDNA genotyping, with about 33% of screened patients receiving a matched targeted agent based on molecular tumor board’s discussions and ESCAT guidance^9^.

### ctDNA validated targets for treatment selection

Large trials of targeted therapies for mBC have demonstrated the clinical utility of ctDNA for detecting specific tumor aberrations and selecting treatments, leading to integration of certain ctDNA assays into standard clinical practice.

Mutations of *PIK3CA* represent the second most common single molecular alteration isolated in mBC following *TP53* SNVs, and are identified in 28%-46% of HR+/HER2- mBC cases^10,11^. The SOLAR-1 trial demonstrated that the combination of the α-selective PI3K inhibitor alpelisib with fulvestrant significantly improves progression-free survival (PFS) over placebo + fulvestrant in HR+/HER2- mBC progressed to first-line endocrine therapy (ET) bearing *PIK3CA* mutations. No PFS benefit was observed in the *PIK3CA*-wild type group, as well as no statistically significant impact on overall survival (OS) in either group at a median follow-up of 42.4 months^11,12^. Patients were enrolled in the *PIK3CA*-mutated cohort based on the identification on tumor tissue of one of 11 *PIK3CA* mutations by the Therascreen test^11^. However, a subsequent subgroup analysis demonstrated a relevant OS improvement for patients with *PIK3CA*-mutant ctDNA, irrespective of their *PIK3CA* testing result on tissue at study screening^11^. In a large study of comprehensive genome profiling (CGP) on mBC tumor tissue and liquid biopsy samples from the Flatiron Health-Foundation Medicine clinico-genomic database, the agreement between FoundationOne CDx on tissue and FoundationOne Liquid CDx on liquid biopsy for *PIK3CA* mutation detection was 77%, increasing to 95% for samples with a cfDNA tumor fraction ≥2%^13^. Moreover, the study showed that 20% of patients with a *PIK3CA* mutation identified by the two CGP panels, testing the whole *PIK3CA* exome either in tumor tissue or ctDNA, had different mutations from the 11 identified by Therascreen. This confirms the previous report by Martìnez-Sàez et al., who suggested that Therascreen misses up to 30% of *PIK3CA*-mutated patients compared to whole-exome sequencing assays^14^. Also, in a phase 2 study of alpelisib monotherapy for HER2- mBC, the addition of ctDNA testing to tissue genotyping increased the number of different *PIK3CA* aberrations detected from 29 to 45 variants^13,15^, highlighting the utility of ctDNA evaluation in improving *PIK3CA* alterations detection, expanding access to alpelisib. Accordingly, the 2022 update to the American Society of Clinical Oncology (ASCO) guidelines on biomarkers in mBC recommends that patients potentially candidate to alpelisib + ET are tested for *PIK3CA* mutations via ctDNA sequencing, with reflex testing on tumor tissue in case of negative ctDNA results^16^.

The *ESR1* mutation has long been known as an acquired resistance mechanism to endocrine treatment with aromatase inhibitors (AIs), by inducing constitutive activation of the estrogen receptor (ER). Virtually absent in untreated mBC, its incidence averages 20-40% in mBCs previously exposed to AIs^17^. Exploratory analyses from the SoFEA and EFECT studies, evaluating the effectiveness of exemestane versus fulvestrant following progression to nonsteroidal AIs in HR+/HER2- mBC, suggested the clinical utility of *ESR1* mutation detection in ctDNA to select patients more likely to benefit from fulvestrant^18–21^. The phase 3 EMERALD trial demonstrated a PFS benefit from treatment with the oral selective estrogen receptor degrader (SERD) elacestrant compared to fulvestrant for patients with *ESR1* mutant mBC progressed to at least one line of ET including a CDK4/6 inhibitor^22^. Based on these results, in January 2023 the US Food and Drugs Administration (FDA) approved elacestrant, together with the Guardant360 CDx companion diagnostic for *ESR1* mutation detection in ctDNA. More recently, the Committee for Medicinal Products for Human Use (CHMP) of the European Medicines Agency (EMA) issued a positive opinion for elacestrant in the same indication. Since June 2023, ASCO recommends *ESR1* mutation testing in blood or tumor tissue obtained at the time of disease progression to ET^23^.

Another potential use of ctDNA is the detection of *BRCA1* and *2* somatic mutations^4^. The PARP inhibitors olaparib and talazoparib have been approved for the treatment of HER2-negative mBC in patients bearing a germline *BRCA1/2* mutation, based on results of OlympiAD and EMBRACA, respectively^24,25^. Even though liquid biopsy has currently no role in selecting mBC patients who could benefit from this treatment, the detection of somatic *BRCA1/2* mutations in ctDNA might have clinical relevance^26^. In fact, there are reports of PARP inhibitors effectiveness in mBC with somatic *BRCA1/2* mutations identified in ctDNA^27^, and phase 2 studies are currently evaluating their possible role as predictive biomarker in this setting (NCT03344965 and NCT03990896). In addition, ctDNA monitoring during treatment with PARP inhibitors can reveal the emergence of resistance via *BRCA2* reversion^28,29^. Moreover, given that routine germline *BRCA1/2* testing in all mBC patients is not yet implemented in several real-world settings, the detection of a somatic *BRCA1/2* mutation in ctDNA could prompt clinicians to search for germline variants^30^.

HER2 amplification can also be estimated in ctDNA, even though with suboptimal detection by available assays, but needs to be confirmed with a validated assay such as fluorescent in situ hybridisation in tissue^4,31^.

Finally, ctDNA has clinical utility in selecting patients for tumor-type agnostic drugs. Based on data from KEYNOTE-158, pembrolizumab obtained FDA and EMA approval for the treatment of solid neoplasms characterized by microsatellite instability-high (MSI-H) status or high tumor mutational burden (TMB-H) defined as ≥10 mutations/megabase, including mBC^32,33^. Moreover, the TAPUR study demonstrated clinically relevant activity of pembrolizumab in heavily pre-treated mBC patients with TMB-H, evaluated either in tumor tissue or ctDNA^34^. While the detection of MSI status in ctDNA has been adequately validated and this can be used for treatment selection, there is currently limited evidence that blood TMB (bTMB) alone can be used for treatment with pembrolizumab^4,35^. Finally, mBC patients might receive additional tumor-agnostic treatments, such as entrectinib and larotrectinib in the presence of *NTRK* gene fusions, or selpercatinib and pralsetinib in case of *RET* fusions; however, these agents target structural variants, with less studied diagnostic yields in ctDNA^4,36–40^.

### Predicting patient benefit from CDK4/6 inhibition

The association of CDK4/6 inhibitors and ET is the preferred first-line treatment for HR+/HER2- mBC, where this approach has demonstrated to improve both PFS and OS^41–44^. Translational analyses from registration studies of CDK4/6 inhibitors evaluated several biomarkers, including ctDNA, for their ability to predict treatment response; however, no validated biomarker has yet been identified to improve patients selection, beyond ER expression in the tumor^45,46^.

An exploratory analysis from PALOMA-3, evaluating fulvestrant +/− palbociclib for HR+/HER2- mBC, demonstrated a worse PFS and OS for patients with either a baseline ctDNA fraction >10%, a *TP53* mutation, or a *FGFR1* amplification, in both treatment arms^47,48^. In contrast, patients with a low circulating tumor fraction (≤10%) seemed to have a greater PFS and OS gain from palbociclib, irrespective of the mutational status of *ESR1*, *PIK3CA* or *TP53*, even though patients harboring these mutations had a worse outcome^48^. Similarly, the presence of ctDNA *PIK3CA* mutation or *ESR1* mutation in baseline plasma samples was not related to the benefit of adding abemaciclib to fulvestrant in patients progressing on previous ET in the MONARCH-2 study^49^. Also in the MONARCH-3 study, evaluating the efficacy of adding abemaciclib to AIs in post-menopausal HR+/HER2- mBC patients, a shorter median PFS was observed for patients harboring one or more of 70 cancer-related gene alterations in baseline ctDNA, regardless of the treatment arm^50^.

Conversely, a recent pooled analysis of MONALEESA-2, −3 and −7, evaluating ET +/− ribociclib in different HR+/HER2- mBC populations, suggested a greater PFS benefit from ribociclib in patients harboring alterations in *ERBB2, FAT3, FRS2, MDM2, SFRP1* and *ZNF217*, while alterations in *ANO1, CDKN2A/2B/2C* and *RB1* seemed to predict decreased sensitivity to ribociclib^51^. Of note, the above results are hypothesis generating, as for none of the above genes a statistical significant interaction with treatment was observed after correction of multiple testing^52^.

In addition, the mutational status of 21 genes in ctDNA was evaluated in exploratory analyses of the PEARL study, which couldn’t demonstrate any superiority of palbociclib + ET compared to capecitabine in the treatment of AI-resistant HR+/HER2- mBC^53^. Comparing plasma samples at baseline and after two weeks of treatment, capecitabine induced a greater extent of ctDNA suppression, and lack of ctDNA suppression was associated with worse outcome in both treatment arms. Also, *TP53* mutations had a poor prognostic value in both patient groups, suggesting an aggressive tumor behavior unrelated to endocrine resistance, while *PIK3CA* mutations apparently conferred a worse OS only to patients treated with palbociclib + fulvestrant^53,54^.

Taken together, these results suggest that ctDNA detection is an unfavorable prognostic biomarker in mBC, likely reflecting a higher tumor burden, while no single molecular aberration can be used in the clinic to predict benefit/resistance from ET+CDK4/6 inhibitors. Further trials are warranted, to validate the findings from the pooled MONALEESA trials.

### Selecting treatment post CDK4/6 inhibitors

At progression to CDK4/6 inhibitors + ET, international guidelines recommend testing for somatic *PIK3CA* and *ESR1* mutations and germline *BRCA1/2* mutations, plus optional *PALB2* testing^16,23,43^. The optimal treatment in this setting should consider the agents previously administered, the duration of response obtained, disease burden, ctDNA genotyping, treatment availability and patient preferences.

In addition, resistance mechanisms might be identified among molecular aberrations isolated in ctDNA. Retinoblastoma (*RB1*) is a tumor suppressor gene, acting downstream in the cyclin D1-CDK4/6-retinoblastoma pathway targeted by CDK4/6 inhibitors. Loss of function of the Rb protein mediates resistance to these agents, as demonstrated by preclinical models and clinical reports^55^. Rb loss is well characterized as a resistance mechanism to CDK4/6 inhibitors, and retrospective biomarker analyses from registration trials confirmed the emergence of *RB1* mutations in 2–9% of exposed patients^55–57^. Recently, the phase 2 MAINTAIN trial provided evidence of a PFS benefit from ribobicilib with a switch in endocrine backbone following disease progression to 1st line ET + CDK4/6 inhibitors^58^. Since most patients were previously treated with palbociclib, this phase 2 trial provided preliminary evidence of the value of switching endocrine therapy and adding ribociclib in patients previously treated with palbociclib. Also, the BioPER phase 2 trial evaluating palbociclib post disease progression (PD) to palbociclib failed to demonstrate a meaningful clinical benefit^59^. Other ongoing phase 3 trials evaluate the role of the new SERD imlunestrant with or without abemaciclib (Ember-3: NCT04975308) or of abemaciclib plus fulvestrant (PostMonarch: NCT05169567) post CDK4/6 inhibitors. The identification of *RB1* alterations in ctDNA should urge clinicians against a rechallenge with CDK4/6 inhibitors.

On the other hand, ctDNA alterations could provide hints on intracellular pathways activity. For instance, besides defining patients eligibility for treatment with elacestrant, the presence of *ESR1* mutations in ctDNA suggests that the disease retains endocrine sensitivity, once constitutive activation of the ER is circumvented by treatment^17^. This concept is also supported by the evidence of a greater PFS benefit from elacestrant in patients with longer response to CDK4/6 inhibition, suggesting a greater endocrine sensitivity of the disease^60^. Also translational analyses from the BOLERO-2 study – demonstrating a shorter PFS and OS for patients with a Y537S or D538G *ESR1* mutation isolated in plasma via droplet-digital PCR – could suggest a reduced efficacy of adding everolimus to exemestane in cases where the disease was driven by a strong constitutive ER activation^61^. Therefore, the detection of *ESR1*-mutant ctDNA after progression to CDK4/6 inhibitors might suggest possible benefit from an endocrine-based second-line treatment.

Moreover, different *ESR1* mutations seem to have variable impact on disease behavior. Both in BOLERO-2 and in plasmaMATCH, different clinical outcomes were observed for patients harboring different *ESR1* variants in ctDNA^61,62^. Translational analyses from plasmaMATCH demonstrated the coexistence in ctDNA of polyclonal alterations of the same gene as well as of molecular alterations known to be mutually exclusive in mBC, with variable clonal dominance^63^. The evidence of divergent tumor evolutionary routes in single patients highlights the relevance of combination approaches to HR+/HER2- mBC treatment, and the potential for ctDNA in directing them.

### Directing an early switch in endocrine backbone

As previously discussed, *ESR1* alterations are acquired in HR+/HER2- BC under the selective pressure of AIs, and predict poor sensitivity to further aromatase inhibition^17^. Given the dynamic and subclonal nature of the alteration, liquid biopsy represents the appropriate tool for *ESR1* mutations detection during ET. Since *ESR1*-mutated tumors might retain sensitivity to SERDs, the PADA-1 study evaluated the efficacy of an early change in ET in case of a rising *ESR1* mutation in the blood of patients treated with palbociclib + AIs for HR+/HER2- mBC^64^. Those patients who presented a new *ESR1* mutation in ctDNA, or an increased level of a known mutation, with no radiologic disease progression, were randomized to continue AI vs switching to fulvestrant, while continuing palbociclib. The median PFS from random assignment was 11.9 months for fulvestrant + palbociclib and 5.7 months for AI + palbociclib^64^. Besides showing the clinical benefit of an early switch in endocrine backbone for these patients, the trial proved the feasibility of serial ctDNA monitoring to track resistance mechanisms and guide treatment in mBC. Applying the same principle, the ongoing SERENA-6 study (NCT04964934) is currently exploring the benefit of a switch from AIs to the oral SERD camizestrant as endocrine backbone to palbociclib upon detection of *ESR1* mutations in ctDNA^65^.

## Integrating ctDNA and imaging response

Plasma ctDNA levels largely depend on tumor burden and tumor cells turnover^66^. Given the short half-life of ctDNA in plasma – about 2 hours – it has the potential to reflect tumor dynamics almost in real time^2^. Currently available data support the assumptions that a fall in ctDNA corresponds to a positive effect of treatment on cancer, and that such measurement can provide an adequate surrogate of the overall aggregated response of a patient to treatment^67^.

In 2013, Dawson et al. demonstrated, in a group of mBC patients treated with chemotherapy (CT) or ET, that ctDNA had a greater correlation with radiologic changes in tumor burden compared to both CA15-3 and circulating tumor cells, also providing a more timely measure of disease response^68^. Another study on 420 patients with metastatic colorectal, ovarian or non-small cell lung cancer treated with CT, evaluated the relationship of ORR and ctDNA response – defined as the fraction of patients converting from measurable ctDNA at baseline to unmeasurable levels at first radiologic evaluation – with OS^69^. ctDNA response outperformed ORR as a predictor of OS, with higher sensitivity and reproducibility. Furthermore, Stover et al. developed an algorithm, ichorCNA, to quantify the cfDNA tumor fraction (TFx) based on 0.1x coverage whole-genome sequencing. Applying this method to 506 plasma samples from 164 patients with advanced TNBC, they observed a significantly worse survival among patients with TFx ≥10%^70^.

Similar evidence came from studies of targeted treatments + ET. Davis et al. collected serial blood samples at baseline and during treatment with CDK4/6 inhibitors + ET from 54 patients, to determine the bTMB and copy-number burden (CNB). They observed a decrease in blood CNB after 15–30 days of treatment, while in over 66% of patients an increase from previous nadir levels preceded radiologic detection of PD by ≥3 months, and in 4 cases by 9 months^71^. Similarly, in a phase 2 study of alpelisib monotherapy for HER2- mBC, levels of mutant *PIK3CA* in ctDNA fell in patients achieving a radiologic response, while rising prior to detection of PD, with a median lead time of 54 days from first rise to overt radiologic progression (range, 1-247 days)^15^. Another retrospective proof-of-concept study on 82 patients evaluated ctDNA fluctuations as a potential biomarker of early PD in mBC^72^. The total variant allele fraction (VAF) was calculated by summing up the VAFs of all mutations detected via Guardant360 in ctDNA at different patient time points – i.e. at the onset of a new line of treatment and during treatment – and dividing these by the number of mutations at each time point, to account for polyclonality. No association was detected between baseline ctDNA levels and radiologic progression, but patients showing a rise in ctDNA during treatment had twice the risk of a PD in their subsequent CT scan and a shorter radiologic PFS. Molecular progression could predict radiologic PD in advance of an average 5.8 weeks (range, 4–12 weeks)^72^. Moreover, in a Belgian study, 45 mBC patients underwent 18F-FGD PET/CT and ctDNA assessment after 14 days of treatment with everolimus + exemestane for mBC: either the absence of a 18F-FDG PET/CT response or the detection of ctDNA were associated with a shorter PFS. The group of patients presenting absence of a 18F-FDG PET/CT response and detection of ctDNA at day 14 had the worst outcome^73^.

Additionally, several studies of CDK4/6 inhibitors demonstrated that ctDNA dynamics over the first month of treatment might inform later outcomes. An exploratory analysis from PALOMA-3 demonstrated that the relative change in *PIK3CA*-mutant ctDNA levels after 15 days on treatment was a strong predictor of PFS, and a fall in ctDNA levels was observed after 15 days of treatment with palbociclib but not with ET + placebo^74^. In a study on 50 patients treated with ET + CDK4/6 inhibitors for HR+/HER2- mBC, targeted NGS for 74 cancer genes was performed on plasma samples taken at baseline and after 4 weeks of treatment. For genetic mutations isolated both at baseline and after one cycle of treatment, the authors calculated the ratio of the VAFs at the two timepoints, obtaining a ratio <1 where the VAF for a specific mutation was lower at C2D1 compared to baseline, and vice versa. Next, they calculated the mean of these ratios for each patient (mVAFR), demonstrating a significant association between individual mVAFR and PFS^75^. Similar results were obtained by Darrigues and colleagues, who applied a tumor-informed approach to evaluate ctDNA mutations at baseline and after 15 and 30 days of treatment with fulvestrant + palbociclib in HR+/HER2- mBC^76^. They observed no correlation between baseline ctDNA levels and PFS, but there was a strong association of ctDNA clearance at day 30 with disease response after 3 months of treatment and PFS^76^.

These studies suggest that early variations in the mutational burden of ctDNA have the potential to predict clinical outcomes already few weeks after the onset of CDK4/6 inhibition. Further evidence is needed to fully understand the predictive value of early ctDNA dynamics.

Similar data are now accumulating for other solid neoplasms^77^, leading to initiatives for the structural integration of ctDNA dynamics into imaging-based Response Evaluation Criteria in Solid Tumors (RECIST) – such as the recently published LB-RECIST criteria^78^ – in order to optimize the detection of disease progression^79^. In the future, ctDNA dynamics could inform clinical decisions in situations where the disease is non-evaluable per RECIST criteria, as in bone-only metastatic disease. While RECIST evaluation criteria are tumor agnostic and to some extent treatment agnostic^80^ (with the exception of immunotherapy^81^), it is likely that ctDNA dynamics could be tumor and treatment dependent. Currently, there are several questions for the role of ctDNA dynamics to predict clinical outcome such as: 1. what is the optimal time to assess ctDNA response? 2. What is the level of ctDNA decrease that is associated with outcome? 3. Will adding ctDNA response to RECIST response provide clinically meaningful information to RECIST that can be cost-effective and scalable to most hospitals? The value of ctDNA response using well-validated assays in addition to standard imaging needs to be evaluated in well-conducted prospective clinical trials.

## Conclusions and future perspectives

The data we presented point to an increasing role of ctDNA evaluation in informing clinical decision-making, with some applications already integrated into clinical care. Thanks to technological advances, the amount of information extracted from a single ctDNA test will increase, providing information beyond SNVs, while the same fidelity of genotyping will be achieved from less genomic material. Recent works used low-coverage whole genome sequencing of cfDNA and applied computational approaches to ctDNA processing in mBC, predicting complex tumor phenotypes such as proliferation status, ER expression, histology^82,83^.

On the other hand, the integration of ctDNA assays into real-world settings is still lagging behind^1^. The implementation of ctDNA testing in clinical practice requires setting up dedicated operating procedures for test prescription, sample collection, handling, and analysis, reporting and interpretation of results. More specifically, the standardized reporting of the results needs to include pre-analytical parameters and evidence-based annotations, to facilitate the translation into evidence-based treatment recommendations^1,4^. Databases ranking molecular aberrations based on their actionability by targeted treatments will also facilitate this process, such as ESCAT, ranking aberrations into class I to IV with decreasing level of evidence, and the OncoKB database developed by the Memorial Sloan Kettering Cancer Center, sorting aberrations into 4 levels of evidence^84–86^. The increased integration of ctDNA evaluation in drug development^87^ will further support its increased use in the clinics. Finally, incorporating ctDNA assays into clinical workflows requires considerable investment, administrative vision and ad hoc training for the involved staff^1^. The increased integration of ctDNA assays in clinical practice will help accelerate the delivery of precision medicine.

### Reporting summary

Further information on research design is available in the Nature Research Reporting Summary linked to this article.

### Supplementary information

Reporting Summary
